# Construction and evaluation of Alzheimer’s disease diagnostic prediction model based on genes involved in mitophagy

**DOI:** 10.3389/fnagi.2023.1146660

**Published:** 2023-03-23

**Authors:** Yongyan Pei, Sijia Chen, Fengling Zhou, Tao Xie, Hua Cao

**Affiliations:** ^1^School of Chemistry and Chemical Engineering, Guangdong Pharmaceutical University, Zhongshan, China; ^2^Department of Neurology, The Third Affiliated Hospital of Naval Medical University, Shanghai, China

**Keywords:** mitophagy, Alzheimer’s disease, biomarkers, diagnostic model, machine learning

## Abstract

**Introduction:**

Alzheimer’s disease (AD) is a common neurodegenerative disease. The concealment of the disease is the difficulty of its prevention and treatment. Previous studies have shown that mitophagy is crucial to the development of AD. However, there is a lack of research on the identification and clinical significance of mitophagy-related genes in AD. Therefore, the purpose of this study was to identify the mitophagy-related genes with the diagnostic potential for AD and establish a diagnostic model for AD.

**Methods:**

Firstly, we download the AD gene expression profile from Gene Expression Omnibus (GEO). Limma, PPI, functional enrichment analysis and WGCNA were used to screen the differential expression of mitophagy-related AD gene. Then, machine learning methods (random forest, univariate analysis, support vector machine, LASSO regression and support vector machine classification) were used to identify diagnostic markers. Finally, the diagnostic model was established and evaluated by ROC, multiple regression analysis, nomogram, calibration curve and other methods. Moreover, multiple independent datasets, AD cell models and AD clinical samples were used to verify the expression level of characteristic genes in the diagnostic model.

**Results:**

In total, 72 differentially expressed mitophagy-related related genes were identified, which were mainly involved in biological functions such as autophagy, apoptosis and neurological diseases. Four mitophagy-related genes (OPTN, PTGS2, TOMM20, and VDAC1) were identified as biomarkers. A diagnostic prediction model was constructed, and the reliability of the model was verified by receiver operating characteristic (ROC) curve analysis of GSE122063 and GSE63061. Then, we combine four mitophagy-related genes with age to establish a nomogram model. The ROC, C index and calibration curve show that the model has good prediction performance. Finally, multiple independent datasets, AD cell model samples and clinical peripheral blood samples confirmed that the expression levels of four mitophagy-related genes were consistent with the results of bioinformatics analysis.

**Discussion:**

The analysis results and diagnostic model of this study are helpful for the follow-up clinical work and mechanism research of AD.

## Introduction

Alzheimer’s disease (AD) is a common neurodegenerative disease, whose clinical features include cognitive functional impairment (decline in memory ability, behavioral ability, and life performance) and insidious onset (Knopman et al., 2021). According to statistics, AD patients are still increasing rapidly every year, which will bring severe challenges to the society. Therefore, the early diagnosis and prevention of AD is very important (Scheltens et al., 2021). At present, the diagnosis of AD is based on relevant behavioral characteristics and brain imaging, but it is still difficult to achieve early diagnosis (Porsteinsson et al., 2021; Mahaman et al., 2022). Therefore, it is necessary to screen and construct more accurate specific biomarkers and diagnostic models for AD. And these biomarkers can also be used as effective therapeutic targets for AD.

Mitophagy is a selective autophagy process, which is a mechanism to maintain mitochondrial quality by eliminating dysfunctional mitochondria, and is crucial for cellular homeostasis (Onishi et al., 2021). Mitochondria are the main source of intracellular reactive oxygen species (ROS), and high ROS will directly interfere with cell integrity. At the same time, mitochondria are also energy factories, and the function of neurons is heavily dependent on mitochondrial function, so mitochondrial dysfunction is closely related to neurodegenerative diseases (Garza-Lombó et al., 2020; Cardanho-Ramos and Morais, 2021; Trigo et al., 2022). Mitophagy can remove defective mitochondria, so mitophagy may be a potential strategy for the prevention and treatment of neurodegenerative diseases. A previous study has shown that reduced mitophagy function is thought to be the cause of many neurodegenerative diseases such as AD, Parkinson’s disease (PD), Huntington’s disease (HD), and amyotrophic lateral sclerosis (ALS) (Cen et al., 2021; Jiao et al., 2022). Studies have also shown that resveratrol can reduce Aβ_1–42_-induced cell death by increasing mitophagy (Wang et al., 2018). Therefore, a comprehensive analysis of the key regulatory genes of mitophagy has the potential of AD diagnostic markers and the construction of corresponding diagnostic models can effectively guide clinical decision-making and provide more treatment options for AD patients. Previous studies have revealed associations between genes associated with autophagy, aging, immunity and ferroptosis, and the diagnosis of AD, but the association between mitophagy genes and AD has not been reported.

The purpose of this study was to analyze the different datasets of AD in Gene Expression Omnibus (GEO) from different angles. Limma, PPI analysis, functional enrichment analysis and weighted correlation network analysis (WGCNA) were used to determine the differential expression of mitophagy-related AD genes. Then, machine learning methods [random forest, univariate analysis, support vector machine (SVM), minimum absolute contraction and selection operation (LASSO) regression, and SVM classification] were used to filter and identify diagnostic markers. Finally, ROC, multivariate regression analysis, nomogram, and calibration curve were used to establish and evaluate the diagnostic model. Furthermore, multiple independent datasets, AD cell models, and AD clinical samples were used to verify the expression levels of genes characteristic of the diagnostic model. In conclusion, our study has clinical significance and provides more candidate markers for the diagnosis and mechanism of AD.

## Materials and methods

### Data acquisition

Four AD-related datasets were downloaded from the GEO database: GSE63060, GSE63061, GSE5281, and GSE122063. Details of the four datasets can be found in Supplementary Table 1. Genes involved in mitophagy were obtained from different databases and literatures. (1) Pathway Unification database^1^: Search for “mitophagy” in the database. (2) GO,^2^ KEGG,^3^ MSigDB v6.2^4^: Search for “MITOPHAGY” and “AUTOPHAGY OF MITOCHONDRION” in the database. (3) Previous literature: Search for “mitophagy” in the PubMed. A total of 137 genes involved in mitophagy were obtained (Supplementary material 1; Chen H. -N. et al., 2019; Xu et al., 2022; Zhang et al., 2022).

### Screening of differentially expressed mitophagy-related gene

The “normalize Between Arrays” function in limma package was used to standardize and normalize the GSE63061 dataset, and then all the protein coding genes in GSE63061 were extracted from the expression profile based on gencode.v22.annotation.gtf file. The protein coding genes were compared with all mitophagy-related genes to obtain overlapping genes. Then limma package (version 3.40.6) was used to analyze the differences expressed of overlapping genes to obtain the differentially expressed mitophagy-related genes (DE-MRGs) between the AD sample group and the control group. The screening criteria were | log2 Fold Change (FC)| >1, false discovery rates (FDR) <0.05, and *p* < 0.05 (Xie et al., 2022). Finally, Sangerbox 3.0^5^ (Shen et al., 2022) was used to display the DE-MRGs as volcano maps and heat maps.

### Functional enrichment analysis of DE-MRGs

With gene annotation in org.Hs.eg.db (version 3.1.0) as background, cluster Profiler (version 3.14.3) in R was used for enrichment analysis, and the minimum gene set=20, *p* < 0.05 and FDR <0.25 were considered statistically significant (Yu et al., 2012). GO analysis included cell composition, biological processes (BP), and molecular function (MF).

### Construction of protein–protein interactions

The interaction network of DE-MRGs was constructed using the STRING v11.5.^6^ Confidence score ≥0.4 (Szklarczyk et al., 2021). Then, Cytoscape v3.8.1 was used to visualize the interaction network. In addition, Cytoscape plugin Molecular Complex Detection (MCODE) was used to select subnetworks with the highest score. Finally, functional enrichment analysis was performed for the genes in the subnetwork.

### Weighted correlation network analysis

All the protein coding genes in GSE122063 were extracted from the expression profile based on gencode.v22.annotation.gtf file. Take the protein coding gene as the background. Firstly, the outlier genes and samples were removed by goodSamplesGenes method in WGCNA package. Then, WGCNA was used to build a scale-free co-expression network. Topological overlap matrix (TOM) was constructed to measure the average network connectivity of each gene after obtaining appropriate β threshold. The genes with similar expression profiles were divided into different modules using the dynamic tree cutting method, and the parameters were min module size=30, deep split=2, merge cut height=0.25. The hierarchical clustering method was used to construct the tree graph. Finally, the correlation between characteristic genes and traits (AD and health) in each module was calculated, and the core modules were screened. The module with the highest correlation and statistical significance was identified as the most critical module for further analysis.

### Establishment and evaluation of diagnostic model of Alzheimer’s disease through least absolute shrinkage and selection operator logistic regression and machine learning

Protein–protein interactions subnetwork gene and core module characteristic gene from WGCNA were compared to obtain overlapping genes. Univariate analysis of the overlapping genes was performed with traits (AD and health) as dependent variables. Select *p* < 0.05 gene for further analysis. In order to verify the accuracy of univariate analysis results, we use random forest algorithm to re-analyze overlapping genes. In the random forest algorithm, the number of trees was set to 500. the random algorithm was used to sort genes according to the average decline of Gini index. The Random Forest in R software was adopted to establish the random forest classification model. The key genes were obtained by combining the results of the two algorithms. Then, LASSO logical regression analysis and SVM were used to further verify the key genes. Glmnet package was used for LASSO logical regression analysis. The λ value was determined by cross-validation method. The principle of selecting λ value is to minimize the mean square error of Lasso model. The model variables were determined by λ and regression coefficient plots, in which the variables with zero standardization coefficient can be considered to be eliminated by the Lasso regression model. SVM was carried out with e1071 package, and a classifier was constructed to screen out the core genes, and the receiver operator characteristic (ROC) curve was drawn to evaluate the diagnostic potential of SVM classifier. Finally, the candidate characteristic genes of AD diagnostic model were identified according to the results of LASSO and SVM analysis. Then, multivariate logistic regression analysis was performed on the candidate characteristic genes, and selected the final parameters genes (*p* < 0.05) of the diagnostic model. The diagnostic model formula was: β + α _1_ × expression (gene 1) + α _2_ × expression (gene 1) + …. + α_*n*_ × expression (gene n). Where β was constant and α was the standardized coefficient of logical regression. In order to evaluate the prediction accuracy of the model, we use GSE63061 and GSE122063 as the verification sets and use pROC package to draw receiver operating characteristic (ROC) curve.

### Establishment and evaluation of nomogram

Using rms package, a nomogram model of AD diagnosis was established by integrating the expression levels of all key genes and the age of patients analyzed by logistic regression. In order to evaluate the prediction ability of the model, we calculate the C index and draw a calibration curve to compare the difference between the predicted value and the actual observed value. In addition, GSE63061 was taken as the validation set and the ROC curve was plotted to verify the practicability and reliability of the model.

### Gene set enrichment analysis

Based on GSE63061 expression profile, the related pathways and molecular mechanisms were evaluated from Molecular Signatures Database.^7^ The parameters were set to: minimum gene set=5, maximum gene set=5000, 1,000 resampling times, *p* < 0.05, and FDR <0.25 were considered statistically significant.

### Cells culture

Human neuroblastoma cell line SH-SY5Y was purchased from BeNa Culture Collection (BNCC, China). The culture medium of SH-SY5Y cells was 90% Duchenne Modified Eagle Medium (DMEM, Gibco, USA) + 10% fetal bovine serum (Gibco, USA) + penicillin/streptomycin (100U Maple ml Sigma, USA). The culture condition was 5% CO_2_, 37°C constant temperature incubator. The AD cell model was constructed using Aβ_25–35_ (25 μM, Sigma, China), and the specific steps were referred to previous literature (Xie et al., 2022).

### Real-time quantitative polymerase chain reaction

Peripheral blood samples from 10 AD patients and 10 healthy people (Supplementary material 2) were collected and real-time quantitative polymerase chain reaction (RT-qPCR) was used to detect the expression trend of characteristic genes in the diagnostic model. The Ethics Committee of the Third Affiliated Hospital of Naval Medical University approved the sample collection procedure. Total RNA was extracted from peripheral blood using RNAprep Pure high efficiency total RNA extraction kit (TIANGEN, China). RNA Easy Fast Animal Tissue/Cell Total RNA Extraction Kit (TIANGEN, China) extracts total RNA from cells. RT-PCR was performed using the FastKing one-step RT-PCR kit (TIANGEN, China). The gene primers were designed by primer 5, and the sequences were shown in Supplementary Table 2. The primer was synthesized by Sangon (China). The expression of GAPDH was used as internal control. The relative expression was calculated by 2 ^–ΔΔCt^.

### Statistical analysis

Statistical analysis was performed using SPSS software (version 26.0) and GraphPad Prism (version 8). Student *t*-test was used between the two groups. All data were expressed as mean ± SD. *p* < 0.05 was considered statistically significant.

## Results

### Identification of differentially expressed genes involved in mitophagy in Alzheimer’s disease

**FIGURE 1:**
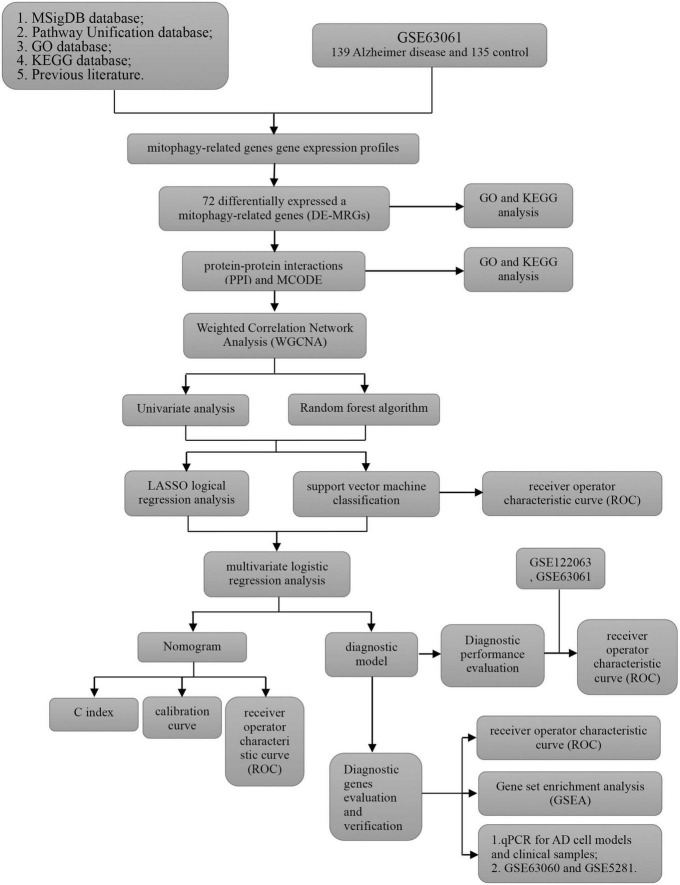
Overview of the research procedure of this study.

**FIGURE 2:**
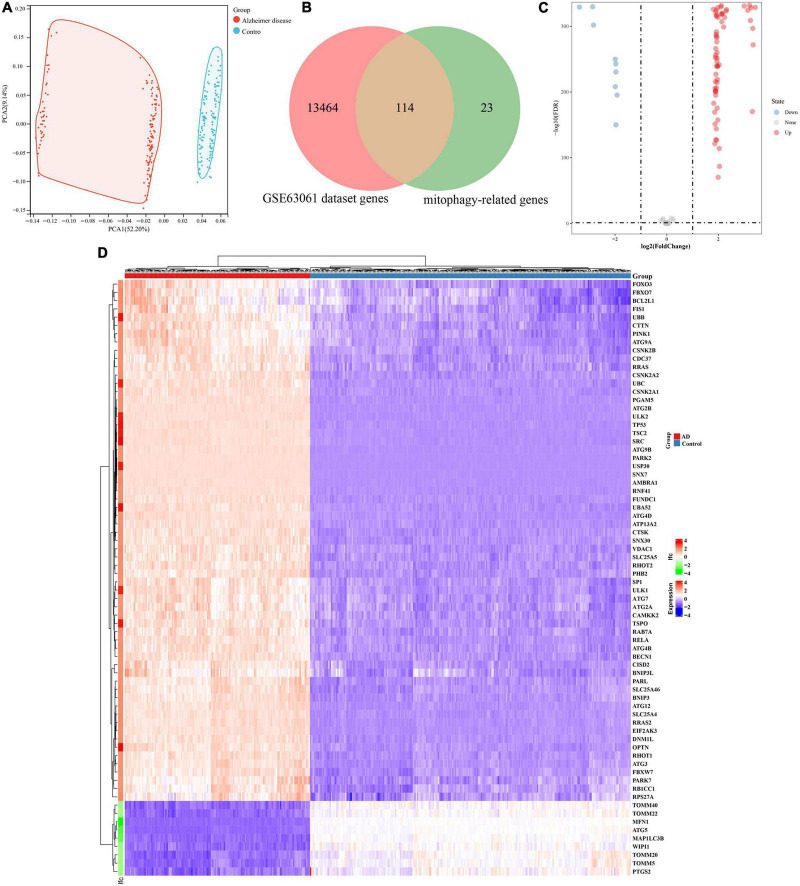
Identification of differentially expressed genes involved in mitophagy in Alzheimer’s disease of GSE63061. **(A)** Principal component analysis of GSE63061. **(B)** Venn showing common genes between GSE63061 dataset and mitochondrial autophagy related genes. **(C)** Volcano of differentially expressed mitophagy-related genes. The red dots represent the significantly upregulated genes and the blue dots represent the significantly downregulated genes. **(D)** Heatmap of the differentially expressed mitophagy-related genes in Alzheimer’s disease and control samples.

The analysis process of this study is shown in Figure 1. GSE63061 contained 13,578 genes. Principal component analysis (PCA) showed that there was a good distinction between AD patient samples and control group samples (Figure 2A). According to the mitophagy database and previous literature, a total of 137 genes involved in the process of mitophagy were obtained (Supplementary material 1). After comparing GSE63061 genes with all mitophagy-related genes, 114 overlapping genes were identified (Figure 2B). Then, according to the differential gene screening criteria, 72 differentially expressed mitophagy-related genes (DE-MRGs) in AD were obtained, including 63 upregulated genes and 9 downregulated genes (Table 1). Volcano and heat maps were used to demonstrate DE-MRGs (Figures 2C, D).

**TABLE 1:** Top 10 (up and downregulated) of differentially expressed mRNAs in Alzheimer disease samples and control group samples of GSE63061.

Symbol	logFC	AveExpr	*t*	*p*-Value
**Upregulation**				
ATG12	2.458483132	6.466785025	27.73996362	1.85E−81
ATG2A	2.530469661	6.93783585	30.82663493	5.86E−91
VDAC1	2.56977612	8.477693809	31.41246822	1.05E−92
PARL	2.796552618	7.159291659	29.41240847	1.14E−86
PINK1	2.951862381	7.32612586	29.30820345	2.38E−86
TSC2	3.267276371	6.369663987	275.4741279	1.40E−87
TSPO	3.282525521	10.16413545	76.99654729	3.93E−237
UBA52	3.310429917	11.51632613	132.1792498	1.85E−81
UBB	3.583647984	12.39026765	25.99957764	7.14E−76
UBC	3.90127177	13.08726982	30.93784185	2.72E−91
**Downregulation**				
TOMM20	−3.91204516	12.83983717	−43.64434194	3.24E−125
MAP1LC3B	−3.84962311	13.86826967	−33.7668447	1.57E−99
TOMM40	−3.82296030	13.1309709	−43.998551	4.67E−126
PTGS2	−3.54614553	12.20801839	−38.45374496	2.76E−112
WIPI1	−3.40558536	11.85569631	−42.14113211	1.36E−121
TOMM5	−3.35246474	11.21257411	−37.84021705	1.12E−110
MFN1	−2.96459138	11.88619632	−20.62264994	1.22E−57
TOMM22	−2.39164620	12.34485273	−24.57755563	3.45E−71
ATG5	−1.69328886	10.99609617	−8.747743475	2.28E−16

### GO and KEGG enrichment analysis of DE-MRGs in Alzheimer’s disease

**FIGURE 3:**
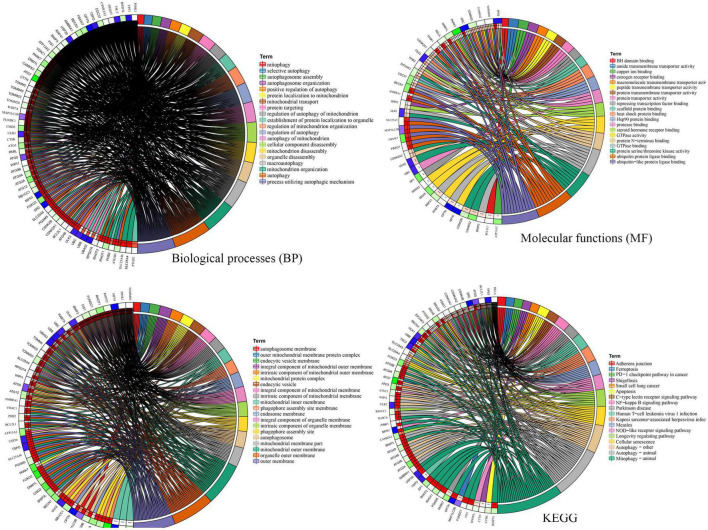
Gene Ontology (GO) and Kyoto Encyclopedia of Genes and Genomes Enrichment analysis of the differentially expressed mitophagy-related genes. BP, biological process; CC, cellular component; MF, molecular function.

To explore the potential functional relationships of these DE-MRGs, we used DAVID to perform functional analysis of DE-MRGs (including GO and KEGG). The results showed that in addition to autophagy related pathways, DE-MRGs were also involved in several BP. BP mainly involve oxygen content regulation, cell apoptosis regulation and protein targeting regulation. MFs mainly include epigenetic regulation and protein binding regulation. In terms of cell components (CC), vacuole membrane, mitochondrial outer membrane, and organelle membrane were the main components. In addition, KEGG results showed that DE-MRGs were mainly enriched in apoptosis, NF-κB signaling pathway, Ferroptosis, Nod-like receptor signaling pathway, and PD (Figure 3).

### Construction of protein–protein interaction network and determination of hub genes

**FIGURE 4:**
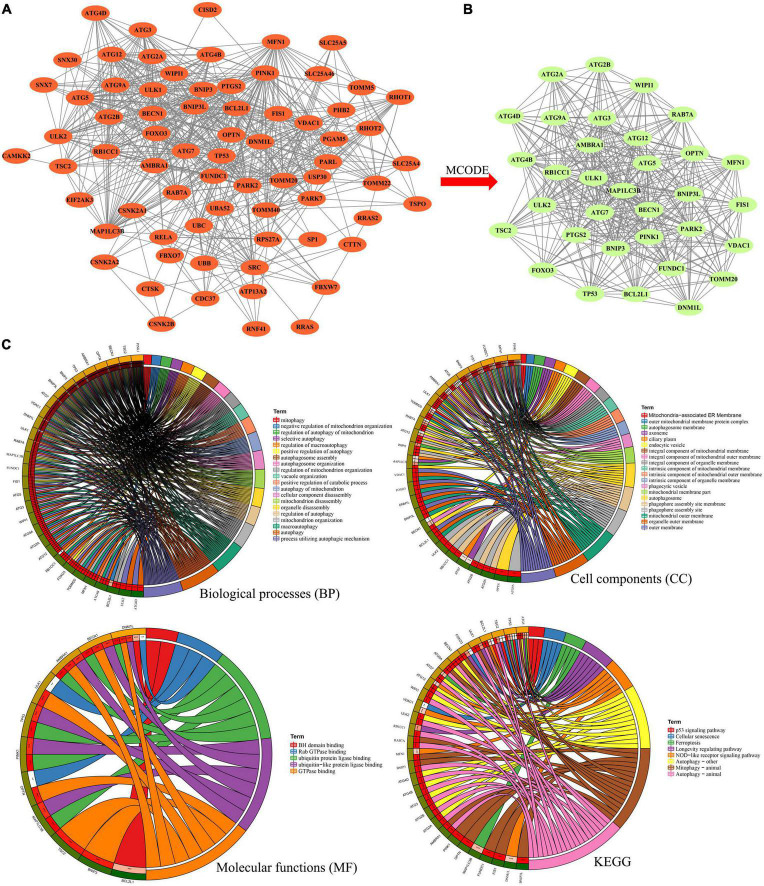
**(A)** Protein–protein interaction network he differentially expressed mitophagy-related genes. **(B)** Sub-network of protein–protein interaction network by MCODE plug-in. **(C)** Gene Ontology (GO) and Kyoto Encyclopedia of Genes and Genomes Enrichment analysis of the hub genes in sub-network. BP, biological process; CC, cellular component; MF, molecular function.

Then, the interactions between DE-MRGs were analyzed by PPI network, which contained 71 nodes and 679 edges, as shown in Figure 4A. In order to further screen the hub genes in PPI, MCODE plug-in was used to further analyze the PPI, and a sub-network with the highest score (score = 24.188) was obtained, comprising 33 nodes and 389 edges (Figure 4B). A total of 33 genes contained in the subnetwork were identified as hub genes. Then 33 hub genes were analyzed by GO and KEGG. In terms of BP, 33 genes were enriched in cellular nutrition, starvation response, oxidative stress, and apoptosis. In terms of CC, they were mainly concentrated in vacuole membrane, synaptic membrane, and organelle membrane. In terms of MF, ubiquitination, GTPase binding, and BH domain binding were mainly involved. In the enrichment analysis of KEGG, they were mainly involved in autophagy, cell senescence, Ferroptosis, Nod-like receptor signaling pathway, and p53 signaling pathway (Figure 4C).

### Construction of weighted gene co-expression network and identification of core modules

**FIGURE 5:**
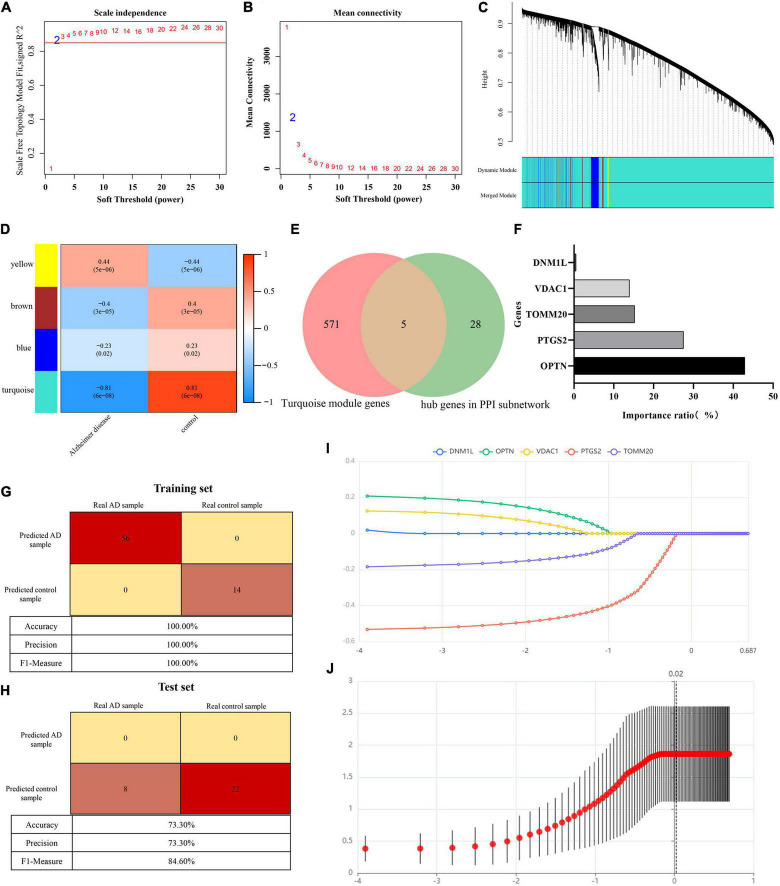
**(A)** Scale-free co-expression network. **(B)** Soft threshold and average connectivity value. **(C)** Dendrogram of all differentially expressed genes clustered. **(D)** Correlations between modules and phenotypes. **(E)** Venn showing common genes between turquoise module genes and hub genes in PPI subnetwork. **(F)** Histogram shows the proportion of importance of random forest features (independent variables). **(G)** Confusion matrix heat map shows the classification results of training sets in GSE122063 by random forest classifier. **(H)** Confusion matrix heat map shows the classification results of test sets in GSE122063 by random forest classifier. **(I)** Result of least absolute shrinkage and selection operator (LASSO) logistic regression algorithm. **(J)** Lasso regression cross validation diagram. Ordinate: model mean square error. Abscissa: the logarithm of λ.

GSE122063 expression profiles (56 AD samples and 44 control samples) were downloaded from GEO database to construct WGCNA. After removing outlier genes and samples, a scale-free co-expression network was constructed. The results show that the scale independence reaches 0.9 when the soft threshold power was confirmed to be 2 (Figure 5A), and the adjacency matrix obtains a relatively high average connectivity value (Figure 5B). Subsequently, four different coexpression modules were completely identified by dynamic tree cutting (Figure 5C). In addition, correlations between modules and phenotypes were analyzed. As shown in Figure 5D, the turquoise module (576 genes) showed the highest correlation with AD (*r* = −0.81, *p* = 6e−08) and was selected for further analysis. Five overlapping genes (DNM1L, OPTN, PTGS2, TOMM20, and VDAC1) involved in mitophagy in AD were identified by comparing the turquoise module genes with 33 hub genes (Figure 5E).

### Establishment and evaluation of Alzheimer’s disease diagnostic prediction model

**FIGURE 6:**
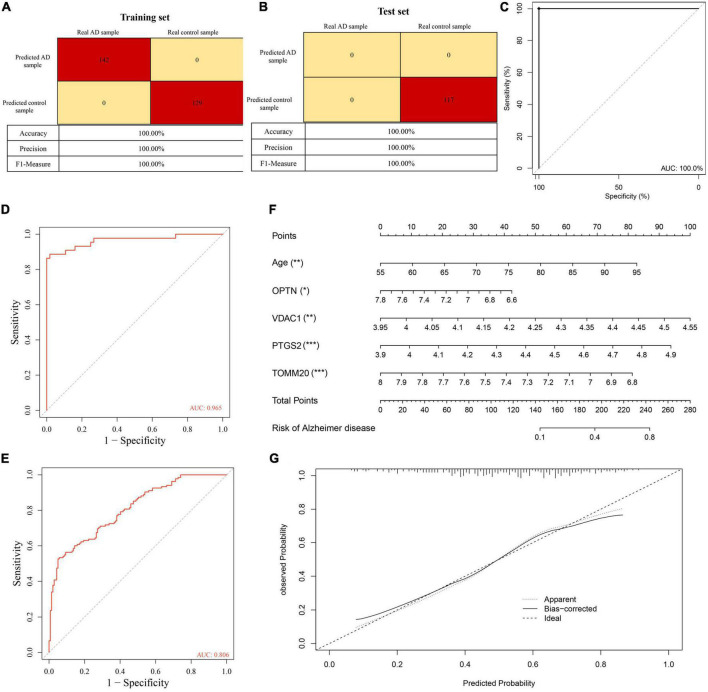
**(A)** Confusion matrix heat map shows the classification results of training sets in GSE122063 by support vector machine (SVM) classification algorithm. **(B)** Confusion matrix heat map shows the classification results of test sets in GSE122063 by SVM classification algorithm. **(C)** The ROC curve verified the feasibility of the diagnosis of the SVM classification algorithm. **(D,E)** The ROC curve verified the feasibility of the diagnosis of the diagnostic prediction model. **(F)** Nomogram visual diagnostic prediction model. **(G)** Calibration curves verify the consistency of nomogram. ***p < 0.001, **p < 0.01, *p < 0.05.

To further understand the role of five overlapping genes involved in mitophagy in the diagnosis and prediction of AD, we expect to screen model characteristic genes from the five overlapping genes to construct a diagnostic prediction model. Firstly, we performed univariate analysis of five genes based on GSE122063 expression profiles. OPTN, PTGS2, TOMM20, and VDAC1 were identified for further analysis (Table 2). The results of random forest algorithm analysis were consistent with univariate analysis. OPTN (42.90%), PTGS2 (27.50%), TOMM20 (15.20%), and VDAC1 (13.90%) were important characteristic genes (Figure 5F), in which the accuracy, accuracy and F1 values of the training set were 100% (Figure 5G). The accuracy and accuracy of the test set were 73.30%, and the F1 value was 84.60% (Figure 5H). Then, we used LASSO regression analysis and SVM classification to verify the above screening results. The results of Lasso regression showed that the four genes (OPTN, PTGS2, TOMM20, and VDAC1) had ideal fit (the optimal sparsity parameter λ was 0.02, *R*^2^ = 0.80, Figures 5I, J). SVM classification algorithm also showed that these four genes had significant classification effects. The accuracy, accuracy and F1 values of the training set and test set were 100% (Figures 6A, B). Moreover, the area under the curve of SVM-ROC was 100% (Figure 6C). These results indicate that OPTN, PTGS2, TOMM20, and VDAC1 are potential markers for the diagnosis of AD. Finally, multivariate logistic regression analysis was conducted for the four genes, and the *p*-values of the four genes were all less than 0.05. The four key DE-MRGs were used to construct a diagnostic prediction model. Table 3 lists the logistic regression coefficients of the four DE-MRGs. The diagnostic prediction formula was *y* = 1.231 + 0.033 × expression (OPTN) + 0.032 × expression (VDAC1)−0.135 × expression (PTGS2)−0.048 × expression (TOMM20). ROC analysis results based on GSE122063 expression profile showed that the area under the curve of the diagnosis and prediction model was 0.965 (Figure 6D), indicating that the model had good prediction ability. Subsequently, the model was used to verify the GSE63061 dataset, and the results showed that the AUC was 0.806 (Figure 6E), which further confirmed the prediction accuracy and stability of the diagnostic model.

**TABLE 2:** Univariate analysis of five genes.

	*B*	SE	Wald	Significance	Hazard ratio	95% confidence interval for the hazard ratio	
						**Lower**	**Upper**
DNM1L	-0.189	0.22	0.742	0.389	0.827	0.538	1.273
OPTN	1.635	0.426	14.734	0.000124	5.128	2.226	11.817
VDAC1	1.403	0.349	16.13	0.000059	4.069	2.051	8.07
PTGS2	-10.413	5.255	3.926	0.048	0.00003	1.01E–09	0.893
TOMM20	-3.323	0.886	14.061	0.000177	0.036	0.006	0.205

**TABLE 3:** Multivariate logistic regression analysis of four genes.

	Unstandardized coefficients		Standardization coefficient	*t*	Significance	95% confidence interval for the hazard ratio		Collinear statistics	
	***B***	**SE**	**β**			**Lower**	**Upper**	**Allowance**	**VIF**
(Constant)	1.231	0.271		4.549	0.000016	0.694	1.768		
OPTN	0.033	0.008	0.217	4.161	0.00007	0.017	0.049	0.725	1.379
VDAC1	0.032	0.013	0.136	2.502	0.014	0.007	0.058	0.669	1.494
PTGS2	-0.135	0.019	-0.54	-7.238	1.17E−10	-0.172	-0.098	0.355	2.817
TOMM20	-0.048	0.02	-0.187	-2.452	0.016	-0.088	-0.009	0.341	2.933

### Evaluation of the characteristics of the diagnostic prediction model

**FIGURE 7:**
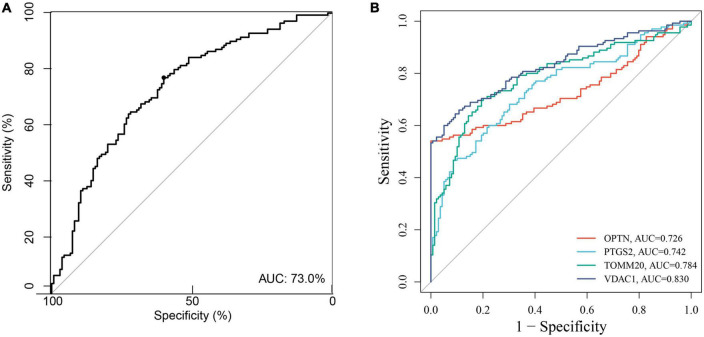
**(A)** The ROC verify the feasibility of the diagnosis of nomogram. **(B)** ROC curve for the four genes in the diagnostic prediction model.

**FIGURE 8:**
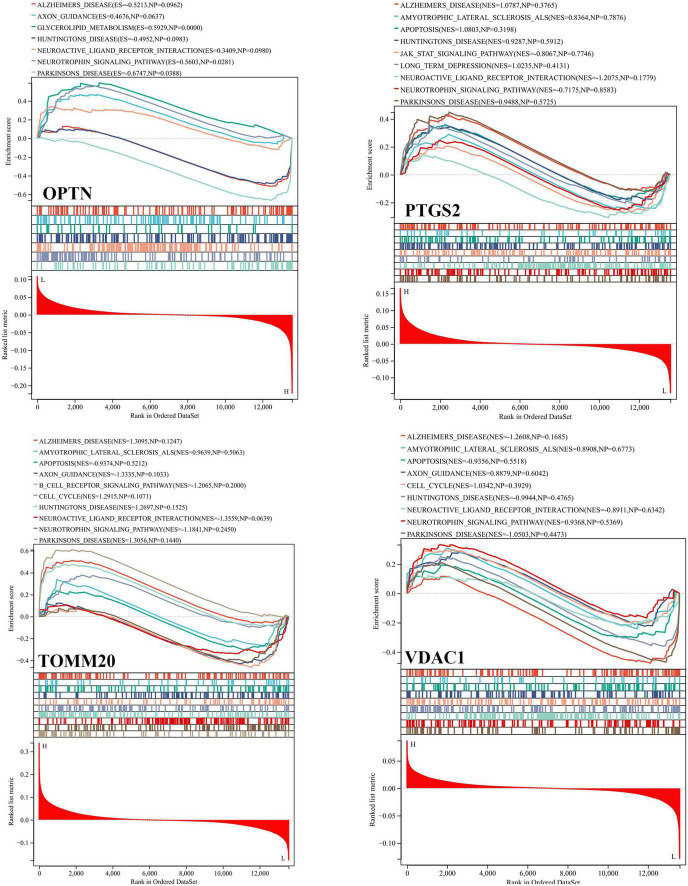
Gene set enrichment analysis (GSEA) of OPTN, PTGS2, TOMM20, and VDAC1 genes using KEGG gene sets.

Based on the GSE63061, we combined OPTN, PTGS2, TOMM20, and VDAC1 genes with age to construct the nomogram corresponding to the diagnosis and prediction model of AD (Figure 6F). The results show that the C index of the nomograph was 0.730, indicating that the model has recognition ability. Subsequently, the nomogram calibration curve for the diagnosis and prediction of AD showed good agreement between the training set and the validation set (Figure 6G). ROC results showed that the AUC of nomograph model was 0.730, indicating that the nomograph model diagnosis model had high feasibility (Figure 7A). In addition, ROC curves were drawn based on the expression levels of OPTN, PTGS2, TOMM20, and VDAC1 genes in the GSE63061. Four DE-MRGs have high diagnostic value for AD. Among the AD samples, VDAC1 showed the highest diagnostic value (AUC = 0.8300), and the areas under the curve of the other three genes were as follows: OPTN (AUC = 0.726), PTGS2 (AUC = 0.742), and TOMM20 (AUC = 0.784) (Figure 7B), suggesting that these four genes have the potential to be diagnostic biomarkers of AD. To further understand the potential biological role of these four genes in AD, we performed gene set enrichment analysis (GSEA) using KEGG gene sets. GSEA results show that these four genes are mainly involved in several neurodegenerative diseases (PD, AD, HD, long-term depression, and ALS), neural function related pathways (neurotrophic signaling pathway, axon guidance, neuroactive ligand receptor interactions, and glycosphingolipid biosynthesis ganglion series), metabolism-related pathways (amino acid metabolism and glucose metabolism), and immune-related pathways (Figure 8).

### Verification of key four DE-MRGs in different datasets, AD cell models, and clinical samples

**FIGURE 9:**
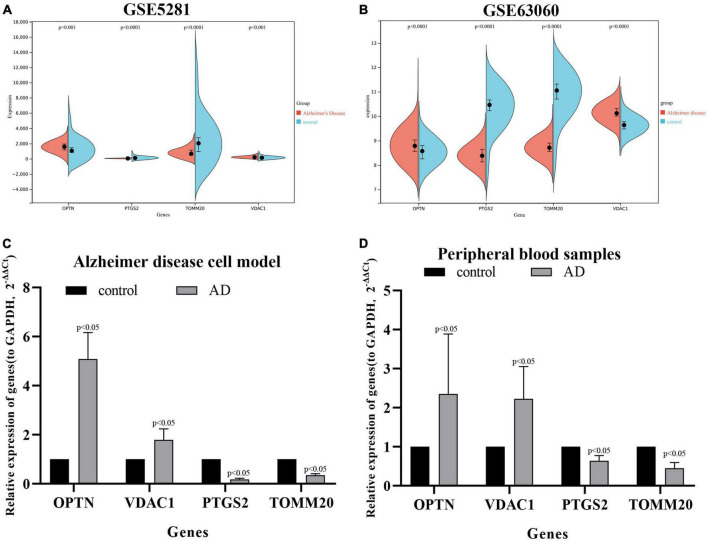
Expression levels of OPTN, PTGS2, TOMM20, and VDAC1 genes in the GSE5281 dataset **(A)**, GSE63060 dataset **(B)**, Alzheimer’s disease cell model **(C)**, and peripheral blood samples of Alzheimer’s disease patients **(D)**.

We download the GSE63060 (peripheral blood samples) and GSE5281 (brain tissue samples) to verify the expression levels of four key DE-MRGs. OPTN, PTGS2, TOMM20, and VDAC1 showed significant differences in both peripheral blood and tissue samples. In AD samples, the expressions of OPTN and VDAC1 were upregulated, while the expressions of PTGS2 and TOMM20 were downregulated (Figures 9A, B). Subsequently, SH-SY5Y cells were treated with Aβ_25–35_(25 μM) to establish AD cell model. The expression levels of OPTN, PTGS2, TOMM20, and VDAC1 in AD cell model were verified by qPCR. The results showed that mRNA expression levels of four genes were consistent with bioinformation analysis (Figure 9C). In addition, to verify the reliability of four key DE-MRGs, peripheral blood samples from 10 AD patients and 10 healthy volunteers were collected for RT-qPCR. The results showed that mRNA expression levels of PTGS2 and TOMM20 in AD group were decreased compared with those in control group (*p* < 0.05). The results of OPTN and VDAC1 were upregulated (*p* < 0.05) (Figure 9D). These results suggest that these four genes have potential as diagnostic and prognostic biomarkers for AD.

## Discussion

Alzheimer’s disease is a common neurodegenerative disease (Kumar and Bansal, 2022), and it has always been the focus of researchers to explore the pathogenic factors and mechanisms of AD. In this process, many hypotheses have emerged, among which the most recognized by researchers is Aβ hypothesis, which believes that Aβ deposition is the most critical pathological change of AD (Ferrari and Sorbi, 2021; Hampel et al., 2021). It has been reported that Aβ deposition in the nervous system can make nerve cells lack of necessary nutrients and apoptosis, leading to nervous system dysfunction (Du et al., 2018; Reddy and Oliver, 2019). The accumulation of Aβ is positively correlated with oxidative stress, while oxidative stress is closely related to mitochondrial dysfunction (Kerr et al., 2017), which also indicates that mitochondrial dysfunction can affect the accumulation process of Aβ. There is evidence that the accumulation of damaged mitochondria is one of the pathogenic factors of many human diseases, including neurodegenerative and cardiovascular diseases, as well as cancer. Mitophagy can eliminate the accumulation of damaged mitochondria and reduce the occurrence of oxidative stress. Among all cell types affected by mitochondrial dysfunction, neurons are susceptible to mitochondrial damage due to their high energy requirements (Shefa et al., 2019). Therefore, mitophagy is a prevention and treatment strategy for neurodegenerative diseases. So, exploring the relationship between mitophagy and AD may find new biomarkers for AD diagnosis and new targets for treatment.

In this study, we combined the genes in the GSE63061 dataset and genes involved in the process of mitophagy, and a total of 72 differentially expressed mitophagy-related genes (DE-MRGs) were screened according to the screening criteria. Subsequently, we further obtained five DE-MRGs through PPI and WGCNA. Then, univariate analysis and random forest classification were used to identify four genes that were closely related to AD. Then, LASSO regression analysis and SVM classification algorithm were used to verify the importance of these four genes in AD. Finally, multivariate logistic regression analysis was used to construct a diagnostic prediction model containing four genes. ROC curve analysis results of test set, training set, and verification set show that the model has good predictive ability. In addition, we combined age and expression levels of these four genes to develop a nomogram model for AD diagnostic. C index, calibration curve, and ROC curve analysis showed that there was a good agreement between the nomogram prediction and the actual observation. According to the investigation, this study is the first to combine mitophagy and differentially expressed genes of AD to establish a diagnostic and predictive model, and to verify and evaluate the accuracy and stability of this model, which may provide an auxiliary role in the diagnosis of AD.

Furthermore, we verified the expression levels of four key genes in different sample types of datasets, AD cell models and peripheral blood of AD patients, and the results were consistent with bioinformatics analysis. Compared with the control group, the expressions of OPTN and VDAC1 were upregulated and the expressions of PTGS2 and TOMM20 were downregulated in AD group. Optineurin (OPTN) is a conserved protein that has been identified as an autophagy receptor and plays a central role in selective autophagy. It has been reported that OPTN, as a receptor, is generally recruited into ubiquitin-coated depolarized mitochondria and has been shown to be a major molecule necessary for mitophagy (Yamano and Youle, 2020). OPTN prevents neurodegeneration by negatively regulating necrosis through degradation of interacting Serine/threonine kinase 1 (RIPK1) receptors (Ames et al., 2021). OPTN has been reported to involve PINK1/Parkin-dependent mitochondrial autophagy. Activated Parkin recruits OPTN proteins into the mitochondria. With the increasing number of OPTN proteins, mitochondrial autophagy is activated (Ke et al., 2022). Some researchers have found that in AD, Aβ exposure can induce the activation of PINK1/Parkin/OPTN pathway, upregulate the expression level of OPTN, and initiate mitochondrial autophagy to reduce the accumulation of Aβ. However, at the later stage, lysosome fusion cholesterol can inhibit this process, and OPTN protein will continue to accumulate in the form of polymerization in the cytoplasm (Roca-Agujetas et al., 2021). Prostaglandin intra peroxidase synthetase 2 (PTGS2) is one of the key enzymes mediating new prostaglandin synthesis and has been shown to play an important role in tumor development (Hashemi et al., 2019). In human brain, PTGS2 is mainly expressed in the cerebral cortex, hippocampus, hypothalamus, and other key parts (Prabhakaran et al., 2021). Thus, PTGS2 may have important implications for behavioral and cognitive function. Xie et al. (2022) reported that PTGS2 can inhibit the proliferation and migration of AD model cells by down-regulating expression, so as to protect nerve cells from injury. However, the specific regulation mechanism is not elaborated in this article. It is reported that PTGS2 also relies on PINK1/Parkin pathway to mediate mitochondrial autophagy. The downregulation of PTGS2 can activate PINK1-PRKN signal to initiate mitochondrial autophagy (Chen Y. et al., 2019), but the correlation mechanism between PTGS2 and the occurrence and progression of AD has not yet been reported. Mitochondrial outer membrane 20 translocation enzyme (TOMM20) is a receptor and a key subunit of the multi-subunit mitochondrial outer membrane (TOM) complex. TOMM20 has been reported to be associated with many malignancies, and increased expression of TOMM20 has been shown to be associated with mitochondrial aggregation (Park et al., 2019). And it has been reported as a marker of mitochondrial autophagy and may be involved in the PINK2/Parkin pathway (Chen et al., 2020). Downregulation of TOMM20 expression is generally associated with mitochondrial morphological and functional impairment, but how autophagy is activated has not been reported. Xiong et al. (2020) reported that valinomycin can improve AD by reducing the level of TOMM20 protein and inducing mitophagy. Voltage-dependent anion channel 1 (VDAC1) is subtype 1 of mitochondrial porin (VDAC). It has been reported that the shape and structure of mitochondria can be regulated through the mitochondrial permeability transition pore to maintain synaptic plasticity (Manczak et al., 2013). The role of VDAC1 in mitochondrial autophagy has been controversial. It has been proposed that VDAC1 may be a part of the PINK1/Parkin pathway in the form of VDAC1/Porin. It has also been proposed that VDAC1 may be superfluous in mitochondrial autophagy activated by PINK1/Parkin (Khalil et al., 2015). The association mechanism of VDAC1 in mitochondrial autophagy remains to be further studied. Shoshan-Barmatz et al. reported that VDAC1 is highly expressed in the brains of AD patients and amyloid precursor protein (APP) transgenic mice, and the expression of this protein may be related to the destruction of neuronal cells. It is suggested that targeting mitochondrial dysfunction through VDAC1 may be a new strategy to inhibit cell death (Shoshan-Barmatz et al., 2018). In summary, on the one hand, the expression levels of OPTN, PTGS2, TOMM20, and VDAC1 in this study were completely consistent with previous studies. On the other hand, OPTN, PTGS2, TOMM20, and VDAC1 genes related to mitophagy were closely associated with AD.

In the past, many diseases diagnosis models or risk models have considered the single factor of differential expression gene, which is difficult to reflect the variation of the disease. Subsequently, some researchers gradually combined some functional process genes to construct corresponding models, which not only narrowed the screening range of model characteristic genes, but further improved the screening specificity. In AD studies, there are selective autophagy related, selective aging related, and selective iron ferroptosis related, but there are no studies related to mitochondrial autophagy. In this study, multiple public datasets were selected for screening, verification and evaluation from different perspectives and forms, which not only avoided the defect of insufficient sample size, but also improved the stability, accuracy, and reliability of the model. In addition, we jointly analyzed the expression levels of four key genes in peripheral blood and brain tissue, and found that there was no difference in the expression trend, indicating that peripheral blood could be used as diagnostic samples of AD patients. However, this study also has some shortcomings: (1) the expression levels of key genes were only verified at the mRNA level, but not at the protein level; (2) with the development of sequencing technology, mitophagy-related genes are still being updated constantly, so the model needs to be updated constantly; (3) the expression levels of key genes were not verified at the animal models.

## Conclusion

In conclusion, four mitophagy-related genes in AD were screened by serial biogenic method and machine learning method, and a logistic regression diagnostic model and a personalized nomogram model based on these genes were constructed. The two models were evaluated and confirmed in different ways to be used in the diagnosis of AD. These models provide a new idea for the prevention and treatment of AD and provide a basis for subsequent studies.

## Data availability statement

The datasets used for analysis in this study (GSE63060, GSE63061, GSE5281, and GSE122063) were derived from the Gene Expression Omnibus (GEO, https://www.ncbi.nlm.nih.gov/geo/), a publicly available repository.

## Ethics statement

This study was approved by the Ethics Committee of the Medical Center of the Third Affiliated Hospital of Naval Medical University. The patients/participants provided their written informed consent to participate in this study.

## Author contributions

YP was responsible for bioinformatics data analysis and writing. SC was responsible for qPCR experiment operation, data analysis, and result writing. FZ performed the cell culture, data correction, and image rendering. TX provided the clinical samples. HC designed the study and approved the article for publication. All authors read and approved the final manuscript.

## Footnotes

1
https://pathcards.genecards.org/

2
http://geneontology.org/

3
https://www.genome.jp/kegg/

4
http://software.broadinstitute.org/gsea/msigdb

5
http://sangerbox.com/

6
https://string-db.org/

7
http://www.gsea-msigdb.org/gsea/downloads.jsp

## Supplementary material

Supplementary Material 1 Mitochondrial autophagy related genes.

Supplementary Material 2 Clinical sample information.
