# Supplementary material for: Construction and evaluation of Alzheimer’s disease diagnostic prediction model based on genes involved in mitophagy
Source: Front Aging Neurosci. 2023 Mar 23;15:1146660. doi: 10.3389/fnagi.2023.1146660 (PMC10077494; doi:10.3389/fnagi.2023.1146660)
Supplement: Supplementary file 1 [file Table_1.docx]

Supplementary table 1 Information of GEO datasets.

| **Dataset** | **Platform** | | **AD** | **Normal** | **Sample type** |
| --- | --- | --- | --- | --- | --- |
| GSE122063 | GPL16699 | Agilent-039494 SurePrint G3 Human GE v2 8x60K Microarray 039381 | 56 | 44 | tissue |
| GSE63060 | GPL6947 | Illumina HumanHT-12 V3.0 expression beadchip | 145 | 104 | peripheral blood |
| GSE63061 | GPL10558 | Illumina HumanHT-12 V4.0 expression beadchip | 139 | 135 | peripheral blood |
| GSE5281 | GPL570 | [HG-U133_Plus_2] Affymetrix Human Genome U133 Plus 2.0 Array | 74 | 87 | tissue |

AD: Alzheimer's disease. **Normal:** **non-dementia controls**
