# Supplementary material for: Construction and evaluation of Alzheimer’s disease diagnostic prediction model based on genes involved in mitophagy
Source: Front Aging Neurosci. 2023 Mar 23;15:1146660. doi: 10.3389/fnagi.2023.1146660 (PMC10077494; doi:10.3389/fnagi.2023.1146660)
Supplement: Supplementary file 2 [file Table_2.docx]

Supplementary table 2. The sequences of primers.

| **Gene symbol** | **Primer** | **Sequences (5’-3’)** |
| --- | --- | --- |
| OPTN | Forward primer | AATGAAGCAAACCATTGCCA |
|  | Reverse primer | GAACAGTAAACTTCCATCTGAGC |
| PTGS2 | Forward primer | AACGCTTTATGCTGAAGCC |
|  | Reverse primer | CCAACTCTGCAGACATTTCC |
| TOMM20 | Forward primer | GGTCACACGGTGAATATGAG |
|  | Reverse primer | GTAAGACCTGCAGTAACTGC |
| VDAC1 | Forward primer | CGAGATTACTGTGGAAGATCAG |
|  | Reverse primer | GATCCTCCCTCTAATAAATATCCAG |
| GAPDH | Forward primer | CATTTCCTGGTATGACAACGA |
|  | Reverse primer | GGGTCTTACTCCTTGGAGG |
